# Benzyl 3-(10-oxo-9,10-dihydrophenanthren-9-ylidene)dithiocarbazate

**DOI:** 10.1107/S160053680904272X

**Published:** 2009-10-28

**Authors:** Qiao-Ru Liu, Song-Mao Chu, Gan-Qing Zhao, Li-Hua Chen, Yong-Jun Han

**Affiliations:** a School of Chemistry and Chemical Engineering, Pingdingshan University, Pingdingshan 467000, People’s Republic of China

## Abstract

In the title compound, C_22_H_16_N_2_OS_2_, the phenanthrene ring is nearly perpendicular to the phenyl ring, making a dihedral angle of 87.2 (2)°. Intra­molecular N—H⋯O inter­actions are present. In the crystal structure, the mol­ecules are linked through inter­molecular C—H⋯O inter­actions. The crystal structure is also stabilized by C—H⋯π inter­actions and weak π–π contacts [centroid-centroid distance = 3.36 (6) Å].

## Related literature

For the biological properties of Schiff bases, see: Bhandari *et al.* (2008 ▶). Recently, some Schiff bases derived from the reaction of *S*-benzyl­dithio­carbazate with aldehydes or ketones have been reported, see: Ali *et al.* (2003*a* ▶,*b* ▶); How *et al.* (2007 ▶); Tarafder *et al.* (2008 ▶); Zhou *et al.* (2002 ▶). For the synthesis of *S*-benzyl­dithio­carbazate, see: Chew *et al.* (2004 ▶). For the synthesis of the title compound, see: Ali *et al.* (2004 ▶).
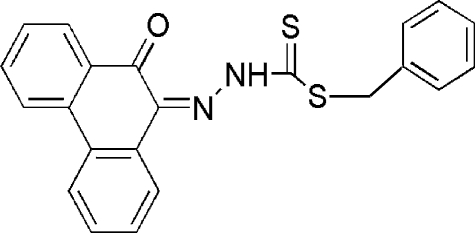


## Experimental

### 

#### Crystal data

C_22_H_16_N_2_OS_2_
*M* *_r_* = 388.49
Monoclinic, 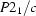

*a* = 14.4945 (19) Å
*b* = 5.6978 (7) Å
*c* = 22.816 (3) Å
β = 93.610 (2)°
*V* = 1880.6 (4) Å^3^
*Z* = 4
Mo *K*α radiation
μ = 0.30 mm^−1^
*T* = 296 K
0.30 × 0.30 × 0.20 mm

#### Data collection

Bruker APEXII CCD area-detector diffractometer
Absorption correction: multi-scan (*SADABS*; Sheldrick, 1996 ▶) *T*_min_ = 0.916, *T*_max_ = 0.943
9220 measured reflections
3316 independent reflections
2638 reflections with *I* > 2σ(*I*)
*R*_int_ = 0.022

#### Refinement

*R*[*F* ^2^ > 2σ(*F* ^2^)] = 0.036
*wR*(*F* ^2^) = 0.098
*S* = 1.06
3316 reflections
244 parameters
H-atom parameters constrained
Δρ_max_ = 0.16 e Å^−3^
Δρ_min_ = −0.22 e Å^−3^

### 

Data collection: *APEX2* (Bruker, 2008 ▶); cell refinement: *SAINT* (Bruker, 2008 ▶); data reduction: *SAINT*; program(s) used to solve structure: *SHELXS97* (Sheldrick, 2008 ▶); program(s) used to refine structure: *SHELXL97* (Sheldrick, 2008 ▶); molecular graphics: *SHELXTL* (Sheldrick, 2008 ▶); software used to prepare material for publication: *SHELXTL*.

## Supplementary Material

Crystal structure: contains datablocks global, I. DOI: 10.1107/S160053680904272X/hg2573sup1.cif

Structure factors: contains datablocks I. DOI: 10.1107/S160053680904272X/hg2573Isup2.hkl

Additional supplementary materials:  crystallographic information; 3D view; checkCIF report

## Figures and Tables

**Table 1:** Hydrogen-bond geometry (Å, °)

*D*—H⋯*A*	*D*—H	H⋯*A*	*D*⋯*A*	*D*—H⋯*A*
N2—H2⋯O1	0.86	1.89	2.560 (2)	134
C12—H12⋯O1^i^	0.93	2.42	3.239 (2)	147
C5—H5⋯*Cg*1^ii^	0.93	2.76	3.559 (2)	144

Symmetry codes: (i) 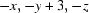; (ii) 
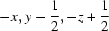
. *Cg*1 is the centroid of the C2–C7 ring.

**Fig. 1.**
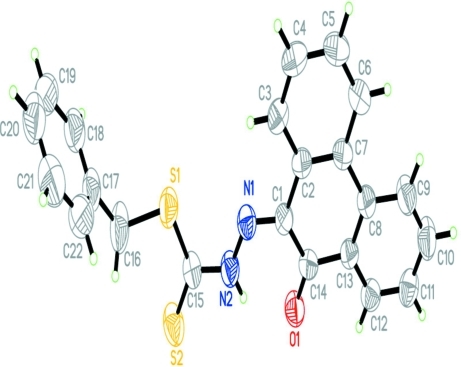
The structure of the title compound, showing 50% probability displacement ellipsoids and the atom-numbering scheme.

**Fig. 2.**
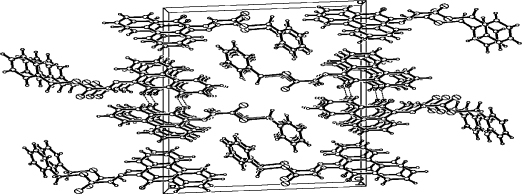
A view of the crystal packing along the b axis. Intermolecular Hydrogen bonds are shown as dashed lines.

## supplementary crystallographic information

### Comment

Schiff bases are versatile compounds which possess excellent biologically properties (Bhandari *et al.*,2008). Recently, some Schiff bases derived from the reaction of *S*-benzyldithiocarbazate with aldehydes or ketones have been reported (Zhou *et al.*, 2002; Ali *et al.*, 2003*a*,b; How *et al.*, 2007; Tarafder *et al.*, 2008). We synthesized the title compound ((Fig. 1)) and report herein its crystal structure.

In the title compound, the bond lengths and angles are comparable to the values in the similar Schiff bases (Zhou *et al.*, 2002). The phenanthrene ring (C1···C14) and dithiocarbazate (N1/N2/S1/S2/C15) fragments lie essentially in the same plane, with a mean deviation from the least-squares plane of 0.0385 Å. The phenanthrene ring (C1···C14) is nearly perpendicular to the phenyl ring (C17···C22) with a dihedral angle of 87.2°.

In the crystal structure, there are intramolecular N—H···O type hydrogen bonds (Table 1). The crystal structure is consolidated by intermolecular C—H···O [3.239 Å] (Fig. 2). It is also stabilized by C—H···Π interactions such as C5—H5···Π (3.642 Å) and C9—H9···Π (3.643 Å) involving phenanthrene ring and phenyl ring of the adjacent molecules respectively. In addition, π-π interactions between the adjacent phenanthrene rings (centroid-centroid distance = 3.36 (6) Å) may also stabilize the crystal packing.

### Experimental

*S*-benzyldithiocarbazate was synthesized as described in the literature (Chew *et al.*, 2004). The title compound was synthesized as described in the literature (Ali *et al.*, 2004). To 9,10-phenanthrenequinone in 60 ml of absolute ethyl alcohol was added a solution of *S*-benzyldithiocarbazate (1.00 mmol) in 20 ml of absolute ethyl alcohol dropwise. The red-brown solution was refluxed for 5.0 h at 353 K. The resultant solution was filtered and left in air for a few days, yielding brown block-like crystals.

### Refinement

In (I), All H atoms were positioned geometrically and refined as riding atoms, with C—H = 0.93 (CH) and 0.97 Å (CH_2_) and *U*_iso_(H) = 1.2*U*_eq_(C), and with N—H = 0.86 Å (NH) and *U*_iso_(H) = 1.2*U*_eq_(N).

### Crystal data

**Table:**

C_22_H_16_N_2_OS_2_	*F*(000) = 808
*M**_r_* = 388.49	*D*_x_ = 1.372 Mg m^−^^3^
Monoclinic, *P*2_1_/*c*	Mo *K*α radiation, λ = 0.71073 Å
Hall symbol: -P 2ybc	Cell parameters from 3545 reflections
*a* = 14.4945 (19) Å	θ = 2.2–27.1°
*b* = 5.6978 (7) Å	µ = 0.30 mm^−^^1^
*c* = 22.816 (3) Å	*T* = 296 K
β = 93.610 (2)°	Block, brown
*V* = 1880.6 (4) Å^3^	0.30 × 0.30 × 0.20 mm
*Z* = 4	

### Data collection

**Table:**

Bruker APEXII CCD area-detector diffractometer	3316 independent reflections
Radiation source: fine-focus sealed tube	2638 reflections with *I* > 2σ(*I*)
graphite	*R*_int_ = 0.022
phi and ω scans	θ_max_ = 25.1°, θ_min_ = 2.2°
Absorption correction: multi-scan (*SADABS*; Sheldrick, 1996)	*h* = −17→12
*T*_min_ = 0.916, *T*_max_ = 0.943	*k* = −6→6
9220 measured reflections	*l* = −27→27

### Refinement

**Table:**

Refinement on *F*^2^	Primary atom site location: structure-invariant direct methods
Least-squares matrix: full	Secondary atom site location: difference Fourier map
*R*[*F*^2^ > 2σ(*F*^2^)] = 0.036	Hydrogen site location: inferred from neighbouring sites
*wR*(*F*^2^) = 0.098	H-atom parameters constrained
*S* = 1.06	*w* = 1/[σ^2^(*F*_o_^2^) + (0.0436*P*)^2^ + 0.4502*P*] where *P* = (*F*_o_^2^ + 2*F*_c_^2^)/3
3316 reflections	(Δ/σ)_max_ < 0.001
244 parameters	Δρ_max_ = 0.16 e Å^−^^3^
0 restraints	Δρ_min_ = −0.22 e Å^−^^3^

### Special details

**Table:**

Geometry. All e.s.d.'s (except the e.s.d. in the dihedral angle between two l.s. planes) are estimated using the full covariance matrix. The cell e.s.d.'s are taken into account individually in the estimation of e.s.d.'s in distances, angles and torsion angles; correlations between e.s.d.'s in cell parameters are only used when they are defined by crystal symmetry. An approximate (isotropic) treatment of cell e.s.d.'s is used for estimating e.s.d.'s involving l.s. planes.
Refinement. Refinement of *F*^2^ against ALL reflections. The weighted *R*-factor *wR* and goodness of fit *S* are based on *F*^2^, conventional *R*-factors *R* are based on *F*, with *F* set to zero for negative *F*^2^. The threshold expression of *F*^2^ > σ(*F*^2^) is used only for calculating *R*-factors(gt) *etc*. and is not relevant to the choice of reflections for refinement. *R*-factors based on *F*^2^ are statistically about twice as large as those based on *F*, and *R*- factors based on ALL data will be even larger.

### Fractional atomic coordinates and isotropic or equivalent isotropic displacement parameters (Å^2^)

**Table:**

	*x*	*y*	*z*	*U*_iso_*/*U*_eq_	
C1	0.12004 (12)	0.9543 (3)	0.10487 (7)	0.0410 (4)	
C2	0.07744 (12)	0.7660 (3)	0.13826 (7)	0.0404 (4)	
C3	0.13280 (14)	0.6058 (3)	0.17087 (8)	0.0505 (5)	
H3	0.1968	0.6203	0.1718	0.061*	
C4	0.09428 (15)	0.4277 (3)	0.20145 (8)	0.0570 (5)	
H4	0.1320	0.3226	0.2230	0.068*	
C5	−0.00057 (15)	0.4045 (3)	0.20025 (8)	0.0551 (5)	
H5	−0.0269	0.2837	0.2209	0.066*	
C6	−0.05593 (14)	0.5600 (3)	0.16850 (7)	0.0496 (5)	
H6	−0.1198	0.5422	0.1679	0.060*	
C7	−0.01904 (12)	0.7447 (3)	0.13706 (7)	0.0413 (4)	
C8	−0.07862 (12)	0.9126 (3)	0.10288 (7)	0.0428 (4)	
C9	−0.17451 (14)	0.8986 (4)	0.10096 (9)	0.0601 (5)	
H9	−0.2023	0.7801	0.1217	0.072*	
C10	−0.22943 (14)	1.0568 (4)	0.06899 (10)	0.0663 (6)	
H10	−0.2934	1.0431	0.0684	0.080*	
C11	−0.19058 (14)	1.2346 (4)	0.03787 (9)	0.0594 (5)	
H11	−0.2279	1.3411	0.0165	0.071*	
C12	−0.09620 (13)	1.2524 (3)	0.03891 (8)	0.0496 (5)	
H12	−0.0693	1.3716	0.0179	0.059*	
C13	−0.04017 (12)	1.0942 (3)	0.07096 (7)	0.0407 (4)	
C14	0.06035 (12)	1.1237 (3)	0.07105 (7)	0.0427 (4)	
C15	0.34732 (13)	1.1176 (4)	0.07602 (8)	0.0513 (5)	
C16	0.51902 (14)	0.9484 (5)	0.11012 (11)	0.0836 (8)	
H16A	0.5363	0.9037	0.0713	0.100*	
H16B	0.5310	1.1148	0.1154	0.100*	
C17	0.57478 (13)	0.8104 (4)	0.15607 (10)	0.0635 (6)	
C18	0.61629 (15)	0.6028 (5)	0.14199 (11)	0.0716 (6)	
H18	0.6081	0.5433	0.1041	0.086*	
C19	0.67014 (17)	0.4821 (5)	0.18405 (14)	0.0816 (7)	
H19	0.6984	0.3422	0.1743	0.098*	
C20	0.68202 (17)	0.5671 (6)	0.23965 (13)	0.0863 (8)	
H20	0.7184	0.4851	0.2677	0.104*	
C21	0.64071 (19)	0.7725 (6)	0.25452 (12)	0.0867 (8)	
H21	0.6489	0.8302	0.2926	0.104*	
C22	0.58678 (16)	0.8938 (5)	0.21272 (12)	0.0777 (7)	
H22	0.5583	1.0329	0.2229	0.093*	
N1	0.20996 (10)	0.9575 (3)	0.10711 (6)	0.0466 (4)	
N2	0.25374 (10)	1.1215 (3)	0.07714 (7)	0.0524 (4)	
H2	0.2225	1.2296	0.0586	0.063*	
O1	0.09460 (9)	1.2850 (2)	0.04354 (6)	0.0577 (4)	
S1	0.39768 (3)	0.88888 (10)	0.11712 (2)	0.06204 (18)	
S2	0.39996 (4)	1.31825 (12)	0.03859 (3)	0.0741 (2)	

### Atomic displacement parameters (Å^2^)

**Table:**

	*U*^11^	*U*^22^	*U*^33^	*U*^12^	*U*^13^	*U*^23^
C1	0.0420 (10)	0.0423 (10)	0.0385 (9)	−0.0001 (8)	0.0004 (7)	−0.0020 (7)
C2	0.0478 (10)	0.0376 (10)	0.0356 (8)	0.0010 (8)	0.0018 (7)	−0.0025 (7)
C3	0.0527 (11)	0.0492 (11)	0.0495 (10)	0.0071 (9)	0.0025 (8)	0.0036 (9)
C4	0.0754 (15)	0.0458 (12)	0.0496 (11)	0.0112 (10)	0.0018 (10)	0.0057 (9)
C5	0.0771 (15)	0.0438 (11)	0.0445 (10)	−0.0062 (10)	0.0052 (10)	0.0042 (8)
C6	0.0570 (11)	0.0479 (11)	0.0439 (10)	−0.0082 (9)	0.0034 (8)	−0.0003 (8)
C7	0.0492 (11)	0.0387 (10)	0.0360 (8)	−0.0024 (8)	0.0016 (7)	−0.0041 (7)
C8	0.0434 (10)	0.0463 (11)	0.0386 (9)	−0.0028 (8)	0.0016 (7)	−0.0045 (8)
C9	0.0489 (12)	0.0670 (14)	0.0646 (12)	−0.0066 (10)	0.0040 (10)	0.0138 (11)
C10	0.0422 (11)	0.0809 (16)	0.0754 (14)	0.0002 (11)	0.0008 (10)	0.0102 (12)
C11	0.0511 (12)	0.0635 (13)	0.0625 (12)	0.0100 (10)	−0.0056 (9)	0.0059 (10)
C12	0.0516 (11)	0.0474 (11)	0.0491 (10)	0.0019 (9)	−0.0015 (8)	0.0040 (9)
C13	0.0439 (10)	0.0404 (10)	0.0374 (9)	−0.0004 (8)	−0.0002 (7)	−0.0031 (7)
C14	0.0489 (10)	0.0405 (10)	0.0384 (9)	−0.0010 (8)	0.0000 (7)	0.0014 (8)
C15	0.0449 (11)	0.0611 (12)	0.0479 (10)	−0.0051 (9)	0.0027 (8)	0.0007 (9)
C16	0.0429 (12)	0.112 (2)	0.0963 (17)	0.0000 (13)	0.0097 (12)	0.0408 (16)
C17	0.0373 (11)	0.0768 (16)	0.0769 (15)	−0.0056 (11)	0.0074 (10)	0.0214 (12)
C18	0.0517 (13)	0.0797 (17)	0.0837 (16)	−0.0092 (12)	0.0081 (11)	0.0086 (13)
C19	0.0622 (15)	0.0702 (16)	0.114 (2)	0.0016 (13)	0.0165 (15)	0.0235 (16)
C20	0.0586 (15)	0.099 (2)	0.100 (2)	−0.0074 (15)	−0.0065 (14)	0.0410 (18)
C21	0.0824 (18)	0.098 (2)	0.0784 (17)	−0.0195 (17)	−0.0051 (14)	0.0110 (16)
C22	0.0680 (16)	0.0705 (16)	0.0959 (19)	−0.0032 (13)	0.0151 (14)	0.0108 (14)
N1	0.0442 (9)	0.0510 (9)	0.0446 (8)	−0.0017 (7)	0.0026 (7)	0.0025 (7)
N2	0.0441 (9)	0.0563 (10)	0.0567 (9)	−0.0018 (8)	0.0021 (7)	0.0118 (8)
O1	0.0509 (8)	0.0552 (8)	0.0664 (8)	−0.0035 (7)	0.0000 (6)	0.0211 (7)
S1	0.0432 (3)	0.0684 (4)	0.0752 (4)	0.0020 (3)	0.0088 (2)	0.0171 (3)
S2	0.0586 (4)	0.0827 (4)	0.0809 (4)	−0.0146 (3)	0.0027 (3)	0.0257 (3)

### Geometric parameters (Å, °)

**Table:**

C1—N1	1.301 (2)	C12—H12	0.9300
C1—C2	1.474 (2)	C13—C14	1.467 (2)
C1—C14	1.480 (2)	C14—O1	1.235 (2)
C2—C3	1.398 (2)	C15—N2	1.358 (2)
C2—C7	1.402 (2)	C15—S2	1.6432 (19)
C3—C4	1.370 (3)	C15—S1	1.739 (2)
C3—H3	0.9300	C16—C17	1.505 (3)
C4—C5	1.380 (3)	C16—S1	1.808 (2)
C4—H4	0.9300	C16—H16A	0.9700
C5—C6	1.371 (3)	C16—H16B	0.9700
C5—H5	0.9300	C17—C18	1.374 (3)
C6—C7	1.399 (2)	C17—C22	1.378 (3)
C6—H6	0.9300	C18—C19	1.382 (3)
C7—C8	1.478 (2)	C18—H18	0.9300
C8—C9	1.390 (3)	C19—C20	1.359 (4)
C8—C13	1.401 (2)	C19—H19	0.9300
C9—C10	1.380 (3)	C20—C21	1.367 (4)
C9—H9	0.9300	C20—H20	0.9300
C10—C11	1.378 (3)	C21—C22	1.380 (3)
C10—H10	0.9300	C21—H21	0.9300
C11—C12	1.371 (3)	C22—H22	0.9300
C11—H11	0.9300	N1—N2	1.341 (2)
C12—C13	1.390 (2)	N2—H2	0.8600
			
N1—C1—C2	116.18 (15)	C12—C13—C14	118.28 (16)
N1—C1—C14	124.21 (16)	C8—C13—C14	120.78 (15)
C2—C1—C14	119.60 (15)	O1—C14—C13	121.05 (16)
C3—C2—C7	119.49 (16)	O1—C14—C1	120.66 (16)
C3—C2—C1	120.34 (16)	C13—C14—C1	118.29 (15)
C7—C2—C1	120.17 (15)	N2—C15—S2	119.68 (15)
C4—C3—C2	121.05 (18)	N2—C15—S1	112.82 (14)
C4—C3—H3	119.5	S2—C15—S1	127.49 (12)
C2—C3—H3	119.5	C17—C16—S1	108.87 (15)
C3—C4—C5	119.87 (18)	C17—C16—H16A	109.9
C3—C4—H4	120.1	S1—C16—H16A	109.9
C5—C4—H4	120.1	C17—C16—H16B	109.9
C6—C5—C4	119.87 (18)	S1—C16—H16B	109.9
C6—C5—H5	120.1	H16A—C16—H16B	108.3
C4—C5—H5	120.1	C18—C17—C22	119.0 (2)
C5—C6—C7	121.82 (18)	C18—C17—C16	120.7 (2)
C5—C6—H6	119.1	C22—C17—C16	120.3 (2)
C7—C6—H6	119.1	C17—C18—C19	120.2 (2)
C6—C7—C2	117.90 (16)	C17—C18—H18	119.9
C6—C7—C8	121.88 (16)	C19—C18—H18	119.9
C2—C7—C8	120.22 (15)	C20—C19—C18	120.3 (3)
C9—C8—C13	117.11 (17)	C20—C19—H19	119.9
C9—C8—C7	121.96 (17)	C18—C19—H19	119.9
C13—C8—C7	120.92 (15)	C19—C20—C21	120.3 (3)
C10—C9—C8	121.44 (19)	C19—C20—H20	119.8
C10—C9—H9	119.3	C21—C20—H20	119.8
C8—C9—H9	119.3	C20—C21—C22	119.7 (3)
C11—C10—C9	120.78 (19)	C20—C21—H21	120.2
C11—C10—H10	119.6	C22—C21—H21	120.2
C9—C10—H10	119.6	C17—C22—C21	120.5 (3)
C12—C11—C10	119.03 (19)	C17—C22—H22	119.7
C12—C11—H11	120.5	C21—C22—H22	119.7
C10—C11—H11	120.5	C1—N1—N2	119.66 (15)
C11—C12—C13	120.71 (18)	N1—N2—C15	120.22 (16)
C11—C12—H12	119.6	N1—N2—H2	119.9
C13—C12—H12	119.6	C15—N2—H2	119.9
C12—C13—C8	120.93 (16)	C15—S1—C16	100.92 (10)

### Hydrogen-bond geometry (Å, °)

**Table:**

*D*—H···*A*	*D*—H	H···*A*	*D*···*A*	*D*—H···*A*
N2—H2···O1	0.86	1.89	2.560 (2)	134
C12—H12···O1^i^	0.93	2.42	3.239 (2)	147
C5—H5···Cg1^ii^	0.93	2.76	3.559 (2)	144

Symmetry codes: (i) −*x*, −*y*+3, −*z*; (ii) −*x*, *y*−1/2, −*z*+1/2.

## References

[bb1] Ali, M. A., Mirza, A. H., Nazimuddin, M., Ahmed, R., Gahan, L. R. & Bernhardt, P. V. (2003*a*). *Polyhedron*, **22**, 1471–1479.

[bb2] Ali, M. A., Mirza, A. H., Ravoof, T. B. S. A. & Bernhardt, P. V. (2004). *Polyhedron*, **23**, 2031–2036.

[bb3] Ali, M. A., Mirza, A. H., Voo, C. W., Tan, A. L. & Bernhardt, P. V. (2003*b*). *Polyhedron*, **22**, 3433–3438.

[bb4] Bhandari, S. V., Bothara, K. G., Raut, M. K., Patil, A. A., Sarkate, A. P. & Mokale, V. J. (2008). *Bioorg. Med. Chem.* **16**, 1822–1831. 10.1016/j.bmc.2007.11.014 18248993

[bb5] Bruker (2008). *APEX2* and *SAINT* Bruker Axs Inc., Madison, Wisconsin, USA.

[bb6] Chew, K. B., Tarafder, M. T. H., Crouse, K. A., Ali, A. M., Yamin, B. M. & Fun, H. K. (2004). *Polyhedron*, **23**, 1385–1392.

[bb7] How, F. N.-F., Watkin, D. J., Crouse, K. A. & Tahir, M. I. M. (2007). *Acta Cryst.* E**63**, o3023–o3024.

[bb8] Sheldrick, G. M. (1996). *SADABS* University of Göttingen, Germany.

[bb9] Sheldrick, G. M. (2008). *Acta Cryst.* A**64**, 112–122. 10.1107/S0108767307043930 18156677

[bb10] Tarafder, M. T. H., Crouse, K. A., Islam, M. T., Chantrapromma, S. & Fun, H.-K. (2008). *Acta Cryst.* E**64**, o1042–o1043. 10.1107/S1600536808013354 PMC2961473 21202563

[bb11] Zhou, J. H., Wang, Y. X., Chen, X. T., Song, Y. L., Weng, L. H. & You, X. Z. (2002). *Chin. J. Inorg. Chem.* **5**, 533–536.

